# The Role of mTOR Signaling as a Therapeutic Target in Cancer

**DOI:** 10.3390/ijms22041743

**Published:** 2021-02-09

**Authors:** Nadezhda V. Popova, Manfred Jücker

**Affiliations:** 1 Laboratory of Receptor Cell Biology, Shemyakin-Ovchinnikov Institute of Bioorganic Chemistry, Russian Academy of Sciences, Miklukho-Maklaya Str., 16/10, 117997 Moscow, Russia; npopova@gmail.com; 2 Institute of Biochemistry and Signal Transduction, University Medical Center Hamburg-Eppendorf, Martinistraße 52, 20246 Hamburg, Germany

**Keywords:** mTOR, PI3K, AKT, cancer, mutation, therapy

## Abstract

The aim of this review was to summarize current available information about the role of phosphatidylinositol-3-kinase (PI3K)/AKT/mammalian target of rapamycin (mTOR) signaling in cancer as a potential target for new therapy options. The mTOR and PI3K/AKT/mTORC1 (mTOR complex 1) signaling are critical for the regulation of many fundamental cell processes including protein synthesis, cell growth, metabolism, survival, catabolism, and autophagy, and deregulated mTOR signaling is implicated in cancer, metabolic dysregulation, and the aging process. In this review, we summarize the information about the structure and function of the mTOR pathway and discuss the mechanisms of its deregulation in human cancers including genetic alterations of PI3K/AKT/mTOR pathway components. We also present recent data regarding the PI3K/AKT/mTOR inhibitors in clinical studies and the treatment of cancer, as well the attendant problems of resistance and adverse effects.

## 1. Mechanistic/Mammalian Target of Rapamycin (mTOR)

The mechanistic/mammalian target of rapamycin (mTOR) is a 289-kDa serine/threonine protein kinase of the phosphatidylinositol-3-kinase (PI3K) family-related protein kinases (PIKKs) that, in humans, is encoded by the *MTOR* gene [1,2,3]. Mammals express one mTOR protein that serves as a core component of two multi-subunit complexes, mTOR complex 1 (mTORC1) and mTORC2 [4]. The complexes perform separate functions in the cell: mTORC1 primarily controls cell growth, while mTORC2 participates in the control of cell survival and proliferation [5].

**Figure 1:**
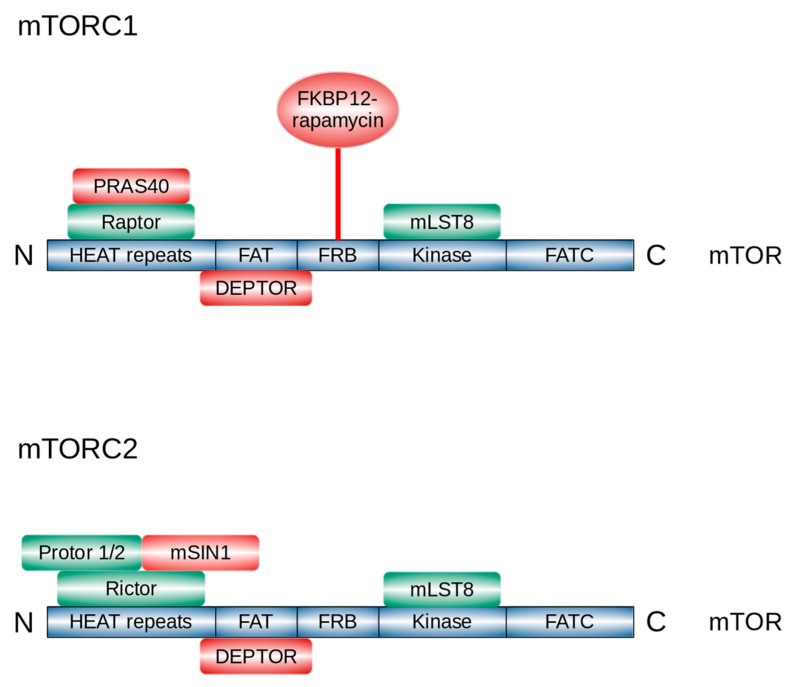
Domain structure of mTOR and components of mTORC1 and mTORC2. DEPTOR, DEP domain-containing mTOR-interacting protein; FAT, FRAP/ATM/TRRAP; FATC, FRAP/ATM/TRRAP/Carboxy terminal; FKBP-12, FK506-binding protein-12; FRB, FKBP12-rapamycin-binding; HEAT, Huntingtin/Elongation factor 3/A subunit of protein phosphatase-2A/TOR1; mLST8, mammalian lethal with SEC13 protein 8; mSIN1, mammalian stress-activated protein kinase interacting protein 1; mTOR, mechanistic target of rapamycin; mTORC1, mTOR complex 1; mTORC2, mTOR complex 2; PRAS40, proline-rich AKT substrate 40 kDa; Protor, protein observed with RICTOR; RAPTOR, regulatory-associated protein of mTOR; RICTOR, rapamycin-insensitive companion of mTOR. The picture was modified from [53].

mTORC1 is composed of three core components: mTOR itself, mammalian lethal with SEC13 protein 8 (mLST8) (also known as G protein β-subunit-like protein GβL [6]), regulatory-associated protein of mTOR (RAPTOR) [7,8], and two non-core components: proline-rich AKT1 substrate 1 (PRAS40) [9] and DEP domain-containing mTOR-interacting protein (DEPTOR) [10] (Figure 1). RAPTOR is fundamental for mTORC1 assembly, stability, correct subcellular localization, and substrate recruitment [7,11,12]. PRAS40 blocks mTORC1 activity until growth factor receptor signaling unlocks PRAS40-mediated mTORC1 inhibition [13].

Like mTORC1, mTORC2 is formed by mTOR and mLST8 [14]. mTORC2 also contains two essential specific components, rapamycin insensitive companion of mTOR (RICTOR) [15] and stress-activated map kinase-interacting protein 1 (mSIN1) [16]; additionally, mTORC2 is associated with the facultative subunit protein observed with RICTOR (Protor)-1/2 [17]. RICTOR is necessary for mTORC2 assembly, stability, and substrate interactions [18]. mSIN1 acts as a negative regulator of mTORC2 kinase activity [16]. Protor-1 may play a role in enabling mTORC2 to efficiently phosphorylate serum and glucocorticoid-activated kinase 1 (SGK1) [19].

Rapamycin allosterically inhibits mTORC1 activity, while mTORC2 demonstrates short-term rapamycin insensitivity [4,20]. The mechanism of rapamycin action remained elusive for more than 20 years after the time of its isolation in 1972. In the 1990s, it was demonstrated that rapamycin acts by binding the peptidyl-prolyl cis-trans isomerase FK506-binding protein 12 (FKBP12) to form a complex that broadly inhibits cell growth and proliferation [21,22]. Biochemical experiments in mammalian cells have revealed that the rapamycin–FKBP12 complex specifically targets and inhibits mTOR [1,2,3]. The rapamycin–FKBP12 complex binds to the FKBP12-rapamycin-binding (FRB) domain on mTOR and inhibits the kinase by directly blocking substrate recruitment and by further restricting active-site access [23].

The structure of mTORC2 obtained by cryogenic electron microscopy showed that RICTOR and mSIN1 together generate steric hindrance, inhibiting the FKBP12–rapamycin complex binding site on mTOR and thereby rendering mTORC2 insensitive to acute inhibition by rapamycin [24,25]. Nonetheless, prolonged rapamycin treatment can inhibit mTORC2 signaling by sequestering the cellular pool of mTOR into rapamycin-bound complexes that cannot nucleate new mTORC2 [26,27].

## 2. Activation of mTOR

### 2.1. PI3K/AKT/mTOR Signalling Pathway

**Figure 2:**
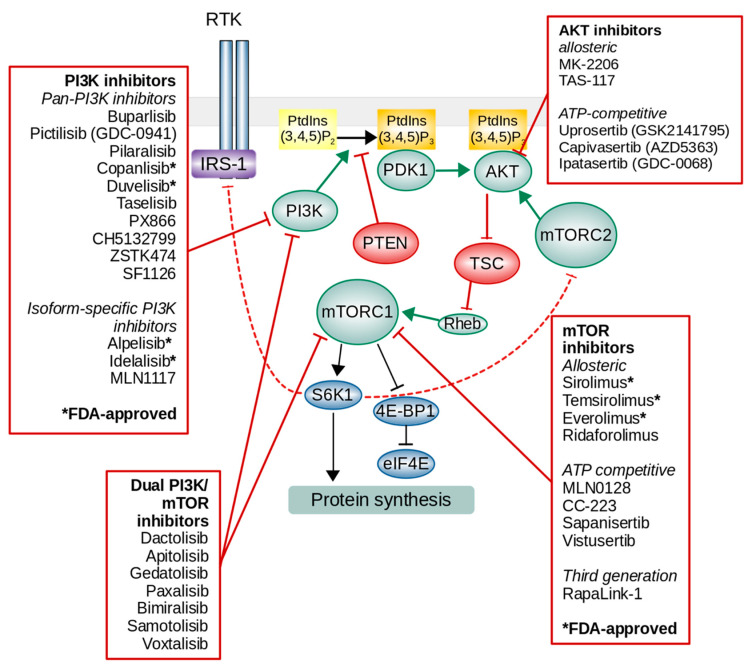
The overview of phosphatidylinositol-3-kinase (PI3K)/AKT/mTOR signaling pathway and inhibitors.

Growth factors, such as insulin or insulin-like growth factor-1 (IGF-1), activate mTORC1 primarily through the stimulation of a pathway involving class I PI3K and its downstream effector AKT [28]. The growth factor binds to extracellular regions of receptor tyrosine kinases (RTKs), and the receptor is activated by ligand-induced receptor dimerization and/or oligomerization [29]. The activation of RTKs results in kinase activation and the autophosphorylation of tyrosine residues in the receptor C-terminal tail (Figure 2). The autophosphorylation of RTKs also recruits and activates a variety of downstream signaling proteins that contain Src homology-2 (SH2) or phosphotyrosine-binding (PTB) domains. These domains bind to specific phosphotyrosine residues within the receptor and engage downstream mediators that propagate critical cellular signaling pathways [30]. PI3K is recruited to the membrane by binding to the phosphotyrosine consensus residues of growth factor receptors or adaptors through one of the two SH2 domains in its adaptor subunit. PI3K generates at the plasma membrane phosphatidylinositol-3,4,5-trisphosphate (PtdIns(3,4,5)P3) from phosphatidylinositol-4,5-bisphosphate (PtdIns(4,5)P2) [31]. A direct antagonist of PI3K is the tumor suppressor phosphatase and tensin homolog (PTEN). PTEN dephosphorylates PtdIns(3,4,5)P3 into PtdIns(4,5)P2 to reverse the activity of PI3K, thereby functioning as an important negative control of incoming signals [32,33]. PtdIns(3,4,5)P3 is a second messenger that recruits phosphoinositide-dependent kinase 1 (PDK1) and AKT to the plasma membrane, and AKT is then phosphorylated by PDK1 at Thr308 [34,35]. To become fully active in response to growth factors, AKT must be further phosphorylated on S473 [36]. While PDK1 catalyzes phosphorylation of T308 [37], mTORC2 phosphorylates AKT at S473 [38,39]. The phosphorylation of S473 on its own is not sufficient to promote AKT activity, but it boosts it by up to ten-fold by promoting a conformational change that stabilizes the active conformation of the kinase domain [40]. AKT phosphorylates several intracellular proteins, including forkhead box O transcription factors (FoxO), BCL2-associated agonist of cell death (BAD) and glycogen synthase kinase 3 (GSK3), to promote cell cycle entry and cell survival. AKT also directly phosphorylates five residues (S939, S981, S1130, S1132, and T1462) on tuberous sclerosis complex 2 (TSC2) within the TSC complex that results in the dissociation of TSC from the lysosomal surface and the activation of Rheb (Ras homolog enriched in the brain) and mTORC1 [41,42,43].

TSC is a heterotrimeric complex that comprises TSC1, TSC2, and TBC1 domain family member 7 (TBC1D7) [44,45]. TSC functions as a GTPase activating protein (GAP) for the small GTPase Rheb [46,47]. TSC stimulates the conversion from the active Rheb-GTP state to the inactive GDP-bound state, and the TSC blockade results in the activation of Rheb. The GTP-bound form of Rheb directly binds the catalytic domain of mTOR and, therefore, activates mTORC1 [9,48]. The activation of mTORC1 occurs via recruitment to the surface of the lysosomes [49], a major hub for the degradation and recycling of macromolecules.

Additionally, AKT activation by growth factors can activate mTORC1 in a TSC-independent manner. AKT phosphorylates mTORC1 inhibitory subunit PRAS40 at T246 that causes PRAS40/RAPTOR dissociation and mTORC1 activation [9,13,50]. TSC can also be inhibited by the TSC2 phosphorylation by the MAP (mitogen-activated protein) kinase extracellular signal-regulated kinase (ERK) [51] and p90 ribosomal S6 kinase (RSK) [52], two downstream substrates of the Ras receptor tyrosine kinase signaling pathway.

Activated mTOR further phosphorylates downstream substrates, thus implicating a variety of cellular processes. mTORC1 directly regulates protein synthesis; plays a central role in lipid and nucleotide synthesis and energetic homeostasis, ribosome biogenesis, nucleotide metabolism, and cell cycle progression; and also negatively regulates catabolic processes such as autophagy, therefore controlling the balance between anabolism and catabolism in response to environmental conditions [53]. mTORC2 mainly regulates cell proliferation, survival, cytoskeletal remodeling, and cell migration [54].

mTORC1 regulates protein synthesis through the phosphorylation of two key molecules, S6K1 (p70 S6 kinase) and 4E-BP1 (eukaryotic initiation factor 4E-binding protein), that promote translation and protein synthesis [55]. Unphosphorylated 4E-BP1 suppresses the initiation of translation by binding and sequestering translation initiation factor 4E (eIF4E). Upon phosphorylation by mTORC1, 4E-BP1 dissociates from eIF4E, allowing for 5′cap-dependent mRNA translation to occur [56,57,58].

S6K1 and mTORC1 upregulate the transcription of rRNA by enhancing the activity of RNA polymerase I and RNA polymerase III through the phosphorylation of the upstream binding factor (UBF) [59], transcription initiation factor 1A (TIF-1A) [60], and MAF1 regulatory factors [61,62]. S6K1 also enhances protein synthesis by activating eIF4B [63], a positive regulator of cap-dependent translation, and by degrading the eIF4A inhibitor PDCD4 (programmed cell death protein 4) [64].

The activation of PI3K-mTOR signaling is normally controlled at numerous levels. Activated RTKs are dephosphorylated by protein tyrosine phosphatases. To control the extent of mTORC1 activation and restore TSC regulation after this stimulus, the mTORC1 substrate S6K1 then directly phosphorylates insulin receptor substrate 1 (IRS-1) as part of a negative feedback loop, blocking the further insulin-mediated activation of the PI3K–AKT pathway [65,66]. S6K1 can also phosphorylate the mTORC2 component mSIN1 on the T86 and T398 sites, which results in the suppression of the mTORC2-mediated activation of AKT [67]. Phosphatidylinositol second messengers at the membrane are dephosphorylated by lipid phosphatases, including PTEN, inositol polyphosphate 4-phosphatase type II (INPP4B), synaptojanin, and Src homology 2 (SH2) domain containing inositol polyphosphate 5-phosphatase (SHIP1/2) [68,69]. PTEN is a dual-specificity phosphatase that selectively removes phosphate from the 3′-hydroxyl group of PtdIns(3,4,5)P3 to produce PtdIns(4,5)P2. Synaptojanins and SHIPs dephosphorylate the 5′-hydroxyl group of PtdIns(3,4,5)P3 to produce PtdIns(3,4)P2. INPP4B removes a phosphate group from the 4′ position of PtdIns(3,4)P2 to produce PtdIns(3)P. The phosphorylation and activation of AKT is also countered by multiple phosphatases including protein phosphatase 2 (PP2A), pleckstrin homology (PH) domain and leucine rich repeat protein phosphatases 1 (PHLPP1), and PHLPP2 [70]. AKT phosphorylation is also inhibited by carboxyl-terminal modulator protein (CTMP), which binds to the AKT carboxyl-terminal regulatory domain [71].

### 2.2. Regulation of mTORC1 by Other Factors

The activity of mTORC1 is also regulated by amino acids, energy status, phosphatidic acid, and oxidative stress (reviewed in [72,73]).

Unlike growth factors, amino acids activate mTORC1 through a PI3K-, AKT-, and TSC-independent mechanism [74,75]. The amino acid-dependent activation of mTORC1 requires the Rag subfamily of Ras small GTPases [76,77]. Amino acids stimulate the loading of RagA or RagB with GTP, which enables RagA/B to interact with the RAPTOR component of mTORC1 [77]. This interaction results in the translocation of mTORC1 to the lysosomal surface, where the Rag GTPases dock on a multi-subunit complex called Ragulator, and lysosomal Rheb stimulates mTORC1 kinase activity [49].

Another important regulator of mTORC1 signaling is the AMP-activated protein kinase (AMPK), the principal energy sensor in most eukaryotic cells [78]. In contrast to growth factors, the depletion of cellular energy inhibits mTORC1 activity and cell growth. During periods of energy or oxygen deprivation, AMP levels rise and promote the activation of AMPK [78,79]. Activated AMPK directly inhibits mTORC1 by phosphorylating RAPTOR [80], and indirectly inhibits mTORC1 by phosphorylating TSC2 on T1227 and S1345, as well as enhancing the ability of TSC2 to inhibit mTOR signaling [81].

An additional regulatory mechanism of mTORC1 signaling involves the lipid second messenger phosphatidic acid (PA) [72]. The potential role of PA in the regulation of mTORC1 signaling was revealed in a study that demonstrated that PA can directly bind to the FRB domain of mTOR and stimulate the activation of the mTOR substrate S6K1 and the phosphorylation of 4E-BP1 in HEK293 cells [82]. It was further demonstrated that phospholipase D (PLD), an enzyme that promotes the hydrolysis of phosphatidylcholine to yield choline and PA [83,84], is a critical regulator in the activation of mTOR signaling by a variety of stimuli [85,86,87]. The elevated expression of either PLD1 or PLD2 activates mTORC1 signaling in various types of cells (reviewed in [72]).

## 3. Deregulation of the PI3K/AKT/mTOR Pathway in Cancer

According to the published data, the PI3K/AKT/mTOR pathway is activated in approximately 70% of ovarian or breast cancers [88,89]. For instance, the aberrant activation of the PI3K/AKT/mTOR pathway has been found in 90% of lung adenocarcinomas (ADCs) and 40% of squamous cell carcinomas (SCCs) [90].

In normal physiology, regulatory mechanisms tightly control the activity and homeostasis of the PI3K/AKT/mTOR pathway, but it can be constitutively activated in various cancers. These mechanisms include the amplification or mutation of genes encoding PI3K subunits, AKT, and other pathway members; the activation of receptor tyrosine kinases, mutation, or overexpression of growth factor receptors, e.g., epithelial growth factor receptor (EGFR) or human epidermal growth factor receptor 2 (HER2); the inactivating mutations in the genes encoding key tumor suppressors PTEN or INPP4B; the inactivating mutations in the genes encoding mTOR regulators such as TSC1 and TSC2; and the activating mutations in *MTOR* itself [91,92,93].

### 3.1. PI3K in Human Cancer

PI3Ks belong to a family of lipid kinases that phosphorylate the 3-OH group of phosphoinositides [94]. Based on their primary structures and in vitro lipid substrate specificity, PI3Ks are classified into three classes: class I PI3Ks, class II PI3Ks, and class III PI3Ks [28]. PI3K-related kinases, which are sometimes referred to as class IV PI3Ks, are protein serine/threonine kinases with a structure similar to the catalytic subunits of PI3Ks. Examples of PI3K-related kinases are mTOR and the DNA-dependent protein kinase (DNA-PK).

Class I PI3Ks are heterodimeric molecules composed of regulatory and catalytic subunits. Class I PI3Ks are further divided into two subclasses—IA and IB—based on their regulatory subunit and upstream activator.

Class IA PI3Ks, activated by receptor tyrosine kinases and RAS [95], consist of a p110 catalytic subunit (p110α, p110β, or p110δ) and one of five p85-like regulatory subunits (p85α, p55α, p50α, p85β, or p55γ). Class IB PI3Ks, activated by G protein-coupled receptors, consist of the catalytic subunit p110γ and one of two regulatory subunits (p101 or p87) [96]. While p110α and p110β are ubiquitously expressed, p110δ and p110γ expression is largely restricted to leukocytes [97].

Class ΙA PI3Ks are widely present in carcinogenic processes. The *PIK3CA* gene, encoding the p110α subunit, is frequently mutated or amplified in the most common human cancers, including those of the breast, colon, gastric, endometrial, cervical, prostate, and lung, as well as glioblastoma [98,99,100,101,102,103]. Most of these mutations occur at two hot-spots—E545K and H1047R—located in the helical domain and the kinase domain of p110α, respectively [103].

Catalytic subunit p110α normally binds to p85, which stabilizes it and controls its enzymatic activity [104]. p110α with the E545K mutation gains the ability to associate with IRS-1 independently of the p85 regulatory subunit, resulting in the constitutive activation of the PI3K pathway and increased cell proliferation, survival, and motility [105]. Another highly recurrent *PIK3CA* mutation, H1047R, increases protein activity through alteration of its catalytic site, resulting in the upregulation of PI3K signaling. However, these two mutants have slightly different phenotypic impact and expression of p110α, as E545K produces a more severe metastatic phenotype than that induced by expressing p110α H1047R in a breast cancer cell line [106].

The *PIK3CA* gene was found to be mutated, on average, in 15% of human cancers, and cancers of the liver, breast, and colon harbor the most *PIK3CA* mutations, with average mutational frequencies of 36%, 26%, and 25%, respectively [99]. A detailed analysis of the COSMIC (Catalogue Of Somatic Mutations in Cancer, UK) database revealed that *PIK3CA* is indeed most frequently mutated in breast cancer (28.83%), especially in estrogen receptor (ER)-positive carcinomas (38.88%), as well as endometrium (27.39%) and urinary tract (20.2%) cancers (Table 1). By contrast, elevated *PIK3CA* expression has been found in about 32% of lung cancers (Table 1).

### 3.2. AKT in Cancers

AKT is an evolutionarily conserved serine/threonine kinase that belongs to the AGC kinase family. Three highly conserved AKT isoforms have been identified: AKT1 (i.e., AKT), AKT2, and AKT3. The alteration of AKT activity is associated with several human diseases, including cancer and diabetes [107]. 

AKT is composed of three conservative structure domains: an N-terminal PH domain, a central kinase domain, and a C-terminal regulatory domain. A single amino acid substitution, E17K, in the lipid-binding PH domain of AKT-1 is a recurrent somatic cell mutation that occurs in breast cancer, meningioma, colorectal, endometrial, and ovarian cancers, and the mutation results in constitutive AKT1 activation [108,109]. This mutation dramatically increases the affinity of E17K AKT to PtdIns(4,5)P2 [110], activates AKT1 by means of pathological localization to the plasma membrane, and stimulates downstream signaling [111,112].

According to the COSMIC database, the frequency of *AKT1* mutations is about 4% in breast cancer and 15% in meningiomas, and elevated expression is more common for endometrium (8.14%) and lung (8.73%) cancers (Table 2).

The overexpression of phosphorylated AKT (p-AKT) is also considered to be an indicator of poor prognosis in many malignancies. A meta-analysis conducted to evaluate the association of p-AKT overexpression with breast cancer prognosis showed that high p-AKT expression was significantly associated with a higher risk of death and disease recurrence [113]. In meta-analyses, p-AKT overexpression was also associated with worse survival in NSCLC (non-small cell lung carcinoma) [114,115], lymph node metastasis and poor prognosis in patients with gastric cancer [116], and poor overall survival and progression-free survival in patients with epithelial ovarian cancer [117].

### 3.3. Alterations of Receptor Tyrosine Kinases

The RTK-mediated activation of the PI3K/AKT/mTOR pathway is crucially important for its oncogenic activity and is clearly linked to the RTK signaling. Examples include PI3K activation by EGFR in lung cancers harboring somatic-activating mutations in *EGFR* [118] and HER2 mutations in breast cancers with HER2 amplification [119]. *EGFR* mutations have also been reported in colorectal cancers [120] and glioblastomas [121].

EGFR/HER1/ErbB1 and HER2/ErbB2 are the members of the ErbB receptor family. Activated receptors bind various signaling proteins and stimulate activation of many signaling pathways, including the PI3K/AKT/mTOR, Ras/RAF/MEK/ERK, phospholipase C (PLC)-γ1, and SRC pathways (reviewed in [122]). It has been shown that many cancer cells are characterized by EGFR hyperactivation, gene amplification leading to receptor overexpression, or mutants with dysregulated signaling [123]. In the *EGFR* gene, mutations occur within exons 18–21, which encode a portion of the EGFR kinase domain. For instance, approximately 80–90% of patients with EGFR-mutated, non–small cell lung cancer have either deletions in exon 19 or substitutions of leucine for arginine (L858R) in exon 21 of the *EGFR* gene [124].

An analysis of the COSMIC database displayed that the incidence of *EGFR* mutations significantly varies among the different types of cancers. The highest frequency is detected in lung cancers (26.58%) and especially in adenocarcinoma (30.61%), where AKT is also overexpressed (Table 3). In contrast to lung cancer, the *EGFR* mutations are rare in breast cancer [125,126], with a frequency of no more than 5–6% (Table 3). However, the overexpression of EGFR is observed in 15–30% of breast carcinomas and is associated with a large tumor size and poor clinical outcomes [126].

### 3.4. PTEN

The PTEN protein consists of two major domains, the N-terminal phosphatase catalytic domain (residues 7–185) and a C-terminal domain (residues 186–351) [127]. The N-terminal phosphatase domain of PTEN contains a consensus PtdIns(4,5)P2-binding motif, and the C-terminal part contains the lipid-binding C2 domain. The C2 domain is believed to be required for the correct positioning of PTEN at the plasma membrane, the site of the lipid substrates of PTEN [128].

PTEN antagonizes the PI3K by dephosphorylating PtdIns(3,4,5)P3 to PtdIns(4,5)P2; therefore, PtdIns(3,4,5)P3 levels are strictly regulated by PTEN in normal cells. PTEN is frequently inactivated in human cancers through several mechanisms, including mutation, a loss of heterozygosity, methylation, the aberrant expression of regulatory microRNA, and protein instability [129,130]. Elevated PtdIns(3,4,5)P3 levels, through the loss of PTEN function, cause the constitutive activation of AKT and downstream cascades such as mTOR signaling [41]. This leads to cell survival, growth, proliferation, and decreased apoptosis [131,132,133].

PTEN has been shown to be lost or inactivated by multiple mechanisms in a wide spectrum of human cancer types: a loss of heterozygosity of PTEN was found in 60–80% of patients with glioblastoma and in 45% of endometrioid carcinoma of the ovary; a loss of PTEN protein expression was found in 20–40% of patients with colorectal cancer; and a decreased PTEN expression was found in 30% of patients with head and neck cancer, PTEN mutations were found in up to 40% of patients with glioblastoma and in 15–50% of patients with prostate cancer [134]. Mutations of the human *PTEN* gene are also frequently observed in breast cancer, glioblastoma, endometrial cancer, malignant melanoma, and prostate cancer [135]. Deletions of PTEN have also been shown in lung cancer. The loss of PTEN expression assessed by immunohistochemistry has been demonstrated in up to 24% of 125 resected early stage NSCLC specimens [136]. Another series of early stage NSCLC specimens revealed that PTEN protein expression was reduced or lost in 74% of tumors [137]. Elevated levels of miR-21 in NSCLC, relative to adjacent non-tumor tissues, were correlated with decreased PTEN mRNA levels and advanced tumor stages [138].

An analysis of the COSMIC database revealed *PTEN* mutations in 55.71% of endometrioid carcinomas, in 39.68% of endometrium cancers, in 13.36% of central nervous system cancers, and in 8.4% of skin cancers (Table 4).

### 3.5. mTOR and Cancer Development

Receptor tyrosine kinase hyperactivation or overexpression, PTEN loss of function, and mutations in *PIK3CA* and *AKT* are not the only mechanisms that can lead to mTOR activation. mTOR can become activated via additional molecular mechanisms that include gene amplification and mutation (reviewed in [139]). It was reported that two different point mutations, S2215Y and R2505P, identified in the COSMIC database, confer the constitutive activation of mTOR signaling in cell cultures even under nutrient starvation conditions. S2215Y was identified in large intestine adenocarcinoma, whereas R2505P was identified in renal cell carcinoma [140]. In cell cultures, the association of the mTOR P2229R mutation with the activation of both the mTORC1 and mTORC2 pathways, increased cell proliferation, and cell survival was also demonstrated [141]. Grabiner et al. identified 33 *MTOR* mutations that occur in multiple cancer types and confer pathway hyperactivation [142]. It was also found that resistant colonies emerged after the exposure of the MCF-7 breast cancer cell line to high concentrations of a first generation mTORC1 inhibitor, rapamycin, or a second generation mTOR ATP competitive inhibitor (AZD8055) for three months. Deep sequencing revealed that the AZD8055-resistant clones harbored an mTOR mutation located in the kinase domain at the M2327I position, while two rapamycin-resistant clones contained mutations located in the FKBP12-rapamycin binding domain at positions A2034V and F2108L [143]. 

An analysis of the COSMIC database revealed an overexpression of MTOR in ovary (9.77%), urinary tract (8.33%), and skin (8.25%) cancers and a downregulation in tumors of the central nervous system (13.06%). The highest incidence of *MTOR* mutations is in meninges (18.63%), endometrium cancer (10.43%), and, especially, endometrioid carcinoma (12.63%) (Table 5). 

Many clinical reports have attempted to investigate the roles and the potential prognostic value of mTOR and p-mTOR in a variety of cancers. A meta-analysis of data from 915 patients with esophageal squamous cell carcinoma (ESCC) was conducted to evaluate the prognostic and clinicopathological significance of mTOR/p-mTOR expression. The pooled analysis identified that positive mTOR/p-mTOR expression was significantly correlated with the worse conditions of differentiation degree, depth of tumor invasion, and lymph node metastasis but had no relationship to gender. Moreover, mTOR/p-mTOR expression was also significantly associated with worse overall survival, disease-free survival, and cancer-specific survival of patients with ESCC [144]. 

The prognostic impact of the PI3K/AKT/mTOR signaling pathway in advanced esophageal squamous cell carcinoma was also assessed in 145 tumor and 145 non-tumor samples of patients from China. The PI3K/AKT/mTOR signaling pathway was shown to be significantly upregulated and PTEN was largely downregulated in tumor tissue. The expression level of PTEN, mTOR, p-mTOR, and S6K1 was closely related to the presence of lymph node metastases. The expression of PTEN, mTOR, and S6K1 were also correlated to the TNM stage and overall survival [145].

## 4. Targeting PI3K/AKT/mTOR

The PI3K/AKT/mTOR signaling pathway plays an important role in cell growth, proliferation, and survival, and it is one of the most commonly deregulated pathways found in human cancers, which makes components of this pathway attractive targets for anticancer therapy.

### 4.1. PI3K Inhibitors

PI3K inhibitors can be subdivided into pan-PI3K inhibitors, isoform-selective PI3K inhibitors, and dual PI3K/mTOR inhibitors [146]. Dozens of PI3K inhibitors have been developed as potential chemotherapeutic drugs (reviewed in [146,147]). The first PI3K inhibitors wortmannin and LY294002 did not reach clinical trials due to the problems with stability, solubility, and toxicity [148], and the development of the PI3K inhibitor as antitumor agent has been a hotspot area since 2006 when ZSTK474, a new pan-class I PI3K inhibitor with less toxic effects, was first presented [149].

Pan-PI3K inhibitors act on each of the four catalytic isoforms of class I PI3K and have, as expected, broad inhibition potential in a number of tumors. However, this broad activity may lead to a higher risks of side effects and toxicities [150]. Until the end of 2020, two pan-PI3K inhibitors, i.e., copanlisib and duvelisib, and two isoform-selective PI3K inhibitors, i.e., idelalisib and alpelisib, were approved for cancer treatment by the Food and Drug Administration (FDA) (Figure 2). 

Copanlisib (Aliqopa), an inhibitor of PI3K, predominantly against the isoforms PI3Kα and PI3Kδ, was approved by the FDA in September 2017 for the treatment of adult patients with relapsed follicular lymphoma who have received at least two prior systemic therapies (https://www.fda.gov/news-events/press-announcements/fda-approves-new-treatment-adults-relapsed-follicular-lymphoma; 14 September 2017).

Another pan-PI3K inhibitor, duvelisib (Copiktra), is an oral dual inhibitor of PI3Kγ and PI3Kδ. It was approved in September 2018 for the treatment of adult patients with relapsed or refractory chronic lymphocytic leukemia (CLL) or small lymphocytic lymphoma (SLL). It was recommended mainly after at least two prior systemic therapies (https://www.fda.gov/drugs/resources-information-approved-drugs/duvelisib-copiktra-verastem-inc-adult-patients-relapsed-or-refractory-chronic-lymphocytic-leukemia; 24 September 2018).

Isoform-selective inhibitors are emerging as next-generation of PI3K inhibitors with improved, precise targeting and reduced toxicity. This class of inhibitors has been developed to target cancers that are connected to one of the PI3K isoforms. The selective inhibition of specific PI3K isoforms may allow for the administration of therapeutic doses of drugs and have fewer toxicities compared to pan-PI3K inhibitors. On the other hand, isoform-specific PI3K inhibitors have the narrowest profile and may require careful patient selection based on potential biomarkers of sensitivity and resistance [151]. It was also shown that the acquired amplification and mutation of *PIK3CA* cause resistance to selective PI3K inhibitors [152]. It was proposed that PTEN loss can lead to clinical PI3Kα inhibitor resistance [153]. 

Idelalisib (Zydelig), a PI3Kδ inhibitor, was approved in July 2014. Zydelig is currently approved by the FDA for the treatment of relapsed chronic lymphocytic leukemia in combination with rituximab and for the treatment of relapsed follicular B-cell non-Hodgkin’s lymphoma or relapsed small lymphocytic lymphoma in patients who have received at least two prior systemic therapies (https://www.fda.gov/drugs/drug-safety-and-availability/fda-alerts-healthcare-professionals-about-clinical-trials-zydelig-idelalisib-combination-other; 14 March 2016).

Alpelisib (Piqray) is a PI3Kα-specific inhibitor, approved by the FDA in May 2019 for use in combination with endocrine therapy fulvestrant for the treatment of hormone receptor (HR)-positive, HER2-negative, PIK3CA-mutated, advanced, or metastatic breast cancer (https://www.fda.gov/news-events/press-announcements/fda-approves-first-pi3k-inhibitor-breast-cancer; 24 May 2019).

### 4.2. AKT Inhibitors

AKT could be a promising target in PI3K/AKT/mTOR pathway-activated tumors as one of the key effector nodes. However, the number of AKT inhibitors that have been explored in clinical trials is less than that of PI3K inhibitors. Most AKT inhibitors in clinical development inhibit AKT 1, 2, and 3 and are therefore termed as pan-AKT inhibitors. There are two distinct classes of AKT inhibitors currently in clinical development: ATP-competitive and allosteric inhibitors. Allosteric inhibitors can prevent the localization of AKT with its PH domain to the plasma membrane, thereby blocking AKT phosphorylation and activation [154]. ATP-competitive inhibitors bind to the active conformation in which the PH domain has swung out from the kinase domain and expose the ATP-binding pocket [155,156]. Allosteric inhibitors are generally better than ATP-competitive inhibitors because they show less toxicity, reduced side-effects, and greater specificity [157].

MK2206, TAS-117, uprosertib (GSK2141795), afuresertib (GSK2110183), capivasertib (AZD5363), and ipatasertib have shown preliminary activity in phase I trials, and they are being tested in phase II trials in a range of tumors as monotherapy or in combination with the MEK inhibitor selumetinib (+MK2206), the MEK inhibitor trametinib (+GSK2141795), docetaxel (+ipatasertib), and paclitaxel (+AZD5363 or ipatasertib). Phase III studies of ipatasertib and capivasertib in combination with various drugs are also ongoing or will begin soon (Table 6).

### 4.3. mTOR Inhibitors

mTORC1 functions as a downstream effector for many frequently mutated oncogenic pathways including the PI3K/AKT and Ras/RAF/MEK/ERK (MAPK) pathways, and mTOR signaling is hyperactive in a range of 40–90% in different tumor entities [158], which makes mTOR an attractive target for cancer therapy. 

#### 4.3.1. The First Generation: Allosteric mTOR Inhibitors

Rapamycin and its analogs (rapalogs) are the first generation of mTOR inhibitors, which selectively inhibit the activity of mTORC1 by binding to FKBP-12 and forming a ternary complex with mTOR. Rapamycin is a macrolide, that is produced by the microorganism *Streptomyces hygroscopicus* and has shown antifungal properties [159]. Shortly after its discovery, immunosuppressive properties were detected, which later led to the establishment of rapamycin as an immunosuppressant [160,161]. In the 1980s, rapamycin was also found to have anticancer activity [162].

Rapamycin is an allosteric inhibitor of mTOR, and it inhibits some of the functions of mTORC1, such as the phosphorylation of the protein kinase S6K1. The clinical use of rapamycin is limited due to its poor water solubility and stability. Thus, several pharmaceutical companies have developed rapamycin analogs with improved pharmacokinetic properties (Figure 2) (Table 7). 

Rapalogs differ in their chemical properties in terms of drug solubility and metabolism. For example, temsirolimus, a prodrug of rapamycin, and ridaforolimus (MK-8669) are water soluble and may be administered intravenously, whereas rapamycin and everolimus display low solubility and are therefore only available for oral formulations [163]. Rapalogs have been undergoing clinical trials for various malignancies and have already been approved by the FDA for the treatment of specific types of cancers (Table 7). 

Though rapamycin inhibits mTOR with a high specificity, its effectiveness is dose-dependent in different contexts. Two mTOR complexes, mTORC1 and mTORC2, have different sensitivities to rapamycin; different doses of rapamycin are needed to suppress mTOR in different cell lines, as well as the phosphorylation of different mTOR substrates, and these properties of rapamycin dosage can be largely attributed to the competition between rapamycin and phosphatidic acid for mTOR (reviewed in [164]). 

While rapamycin inhibits S6K1, it does not fully inhibit 4E-BP1 phosphorylation, thus making it ineffective in blocking cap-dependent translation in most cell types [165]. Phosphorylated 4E-BP1 inhibits pro-oncogenic eIF4E. eIF4E-mediated translation is upregulated in tumors, and blocking this pathway may be crucial to preventing tumor growth in specific cancers [166,167,168]. On the other hand, the inhibition of mTORC1 may lead to the feedback activation of IGF-IR and AKT, which compromise the anti-cancer effect of rapalogs [169]. Rapalogs have proved more cytostatic than cytotoxic, perhaps because they also only partially block 4E-BP-dependent translation and fail to inhibit the pro-survival pathways regulated by mTORC2–AKT [170,171].

The combination of rapamycin with 5-aminoimidazole-4-carboxamide-1-β-4-ribofuranoside (AICAR) was reported to make rapamycin cytotoxic rather than cytostatic at doses that are clinically tolerated [172]. AICAR is a compound that activates AMPK, and a reciprocal regulation of PLD by AMPK and AMPK by PLD was demonstrated, i.e., the suppression of AMPK activity led to an increase in PLD activity and the suppression of PLD activity resulted in elevated AMPK activity [173]. The authors reported that the suppression of PLD activity by AICAR can improve the efficacy of rapamycin for both mTORC1 and mTORC2, and tolerable doses of rapamycin in combination with AICAR suppress both 4E-BP1 and AKT phosphorylation, as well as inducing apoptosis in cancer cells [172].

#### 4.3.2. The Second Generation: ATP-Competitive mTOR Inhibitors

To more completely inhibit mTOR and target both mTORC1 and mTORC2, a number of ATP-competitive mTOR inhibitors have been developed. A second generation of mTOR inhibitors are small molecular ATP analogues that compete with ATP to occupy the kinase active site of mTOR. Differently from rapalogs, these molecules—also called selective mTOR kinase inhibitors (TORKIs)—ensure a complete block of both mTORC1 and 2, thus preventing AKT phosphorylation due to mTORC2 and avoiding the resistance observed in rapalogs.

In the majority of in vitro studies, compared with rapalogs, the ATP-competitive inhibitors have shown significantly higher inhibitory effects. Large scale trials have not yet been conducted to show greater and convincing efficacy than the currently available best treatment options; hence, TORKIs are still not approved by the FDA.

#### 4.3.3. The Third Generation: RapaLink

Third generation mTOR inhibitors were synthesized and investigated to address the treatment resistance issues found in the use of the rapalogs and TORKIs. The new compounds are called Rapalink because they are made by the conjugation of rapamycin and ATP-competitive mTOR inhibitors, and they can bind both to FRB and the mTOR kinase domain [143]. This dual binding may serve to increase affinity and stability, both of which likely contribute to efficacy. Rapalink-1 has shown increased and durable inhibitory action compared to the first and second-generation inhibitors in glioblastoma and follicular lymphoma in vitro and in vivo [174,175,176]. A resistance of cancer cell cultures to rapalogs and TORKIs was shown to be overcome by the use of Rapalink [143].

The effectiveness of Rapalink-1 was also demonstrated in reducing prostate cancer tumor growth using an in vitro organoids assay and ex vivo tumor slice drug assays. The exposure of LAPC9 to Rapalink-1 was found to block mTORC1/2 signaling and reduce the fraction of CD44+ in vitro. Mice treated with Rapalink-1 showed a significantly delayed tumor growth, and cells recovered from the tumors of treated animals showed a marked decrease of CD44 expression [177]. The in vitro and in vivo therapeutic efficacy of Rapalink-1 against renal cell carcinoma (RCC) was evaluated and compared to temsirolimus. Rapalink-1 showed significantly greater effects against proliferation, migration, invasion, and colony formation in RCC cells. RNA sequencing showed that Rapalink-1 suppressed not only the mTOR signaling pathway but also a part of the MAPK signaling pathway, the ErbB signaling pathway, and ATP-binding cassette (ABC) transporters [178]. No large-sample clinical data have been reported for Rapalink.

### 4.4. Dual PI3K/mTOR Inhibitors

Though the inhibition of mTORC1 and mTORC2 can downregulate AKT S473 phosphorylation, mTOR inhibitors may paradoxically enhance the PI3K/PDK1 axis. Thus, an inhibitor targeting both PI3K and mTOR may have better anti-cancer activity compared to targeting mTOR alone [179,180]. 

PI3K and mTOR both belong to the PI3K-related kinase (PIKK) superfamily, and the catalytic isoform of the p110 subunit and mTOR have structural similarities; as a consequence, certain inhibitory compounds target both kinases. Dual PI3K/mTOR inhibitors are active against PI3K isoforms and both mTORC1 and mTORC2, thus targeting three most critical nodes of the same pathway. AKT activation would also be blocked by these inhibitors since PI3K blocking would diminish the production of PtdIns(3,4,5)P3, which acts as a docking site for AKT and PDK1. The inhibition of mTORC2 would also block the feedback activation of AKT. There are some promising trials with dual PI3K/mTOR inhibitors but only in phase 1 or 2 (Table 8). Therefore, more studies are needed to determine if the dual PI3K/mTOR inhibitors would be more effective than mTOR inhibitors.

Another possibility may be the dual inhibition with mTOR and AKT inhibitors to prevent the feedback activation of PI3K/AKT signaling after mTOR inhibition. Indeed, our own studies with a combinatorial treatment of hepatocellular carcinoma and cholangiocarcinoma cells with RAD001 and MK2206 showed synergistic effects on tumor growth in vitro and in xenotransplantation mouse models in vivo [181,182].

## 5. Summary and Conclusions

The discovery of mTOR is a fundamental breakthrough in the understanding of cell growth, metabolism, and diseases. Studies to determine the regulators and effectors of mTOR signaling have revealed multiple networks that interact together to integrate growth factor, nutrient, and nucleotide signaling. The understanding of the critical role of the mTOR pathway in tumorigenesis has driven to the development of a growing list of PI3K/AKT/mTOR inhibitors, though major clinical success has not been achieved.

The long-term use of the same inhibitor in tumors can lead to drug resistance, which is a main challenge in cancer therapy. Resistance can be developed by several mechanisms including the incomplete inhibition of mTORC1 functions, resistance mutations in *MTOR*, the compensatory activation of different pathways because of the mTORC1 inhibition, and the suppression of negative feedback loops (reviewed in [183]). More research to understand the molecular basis of mTOR networks and potential resistance mechanisms in mTOR-targeted cancer therapy are necessary to rationally apply mTOR inhibitors for the effective treatment of cancer.

A second problem comprises the clinical adverse effects associated with PI3K/AKT/mTOR inhibition that include hyperglycemia, hyperlipidemia, bone marrow suppression, pneumonitis, stomatitis, and hepatotoxicity [184]. Severe toxicities associated with PI3K/AKT/mTOR inhibitors may limit the clinical application and approval of these agents. It is necessary to analyze the mechanism leading to the toxicities of PI3K/AKT/mTOR inhibitors that may help to develop optimal prevention and treatment strategies.

High-throughput molecular profiling, including next-generation sequencing, can give insights into the mechanism(s) of intrinsic sensitivity/resistance and the mechanisms of acquired resistance to PI3K/AKT/mTOR inhibitors. Work must be continued to validate predictive biomarkers, which may help to identify the patients who will most likely benefit from treatment with mTOR inhibitors and allow for the better determination of rational combinations of anti-cancer agents.

## Figures and Tables

**Table 1:** Genetic alterations of the *PIK3CA* gene in human cancers. HER: human epidermal growth factor receptor;

Primary Tumor Tissues	Mutations (%) ^1^	Copy Number Variations (%) ^1^		Gene Expression (%) ^1^	
		Gain	Loss	Over	Under
Adrenal gland	1/883 (0.11%)	1/267 (0.37%)	1/267 (0.37%)	1/79 (1.27%)	-
Autonomic ganglia	7/1605 (0.44%)	-	-	-	-
Biliary tract	180/2501 (7.2%)	-	-	-	-
Bone	44/1277 (3.45%)	-	-	-	-
Breast	6559/22748 (28.83%)	27/1492 (1.81%)	-	97/1104 (8.79%)	8/1104 (0.72%)
– ductal carcinoma	1335/5021 (26.59%)	0/315 (0%)	0/315 (0%)	-	-
– ER-positive carcinoma	619/1592 (38.88%)	-	-	-	-
– HER-positive carcinoma	325/1455 (22.34%)	-	-	-	-
– basal (triple-negative) carcinoma	136/932 (14.59%)	-	-	-	-
– ER-PR-positive carcinoma	211/656 (32.16%)	-	-	-	-
Central nervous system	422/5741 (7.35%)	13/1035 (1.26%)	-	38/679 (5.45%)	-
Cervix	347/2530 (13.72%)	61/299 (20.4%)	-	105/307 (34.2%)	7/307 (2.28%)
Endometrium	1277/4662 (27.39%)	24/586 (4.1%)	-	85/602 (14.12%)	-
– endometrioid carcinoma	698/1964 (35.54%)	19/530 (3.58%)	-	71/545 (13.03%)	-
Eye	1/372 (0.27%)	-	1/80 (1.25%)	-	-
Gastrointestinal tract	42/958 (4.38%)	-	-	-	-
Hematopoietic and Lymphoid	110/8604 (1.28%)	1/661 (0.15%)	-	12/221 (5.43%)	3/221 (1.36%)
Kidney	68/3166 (2.15%)	3/995 (0.3%)	1/995 (0.1%)	14/600 (2.33%)	4/600 (0.67%)
Large intestine	3234/22,967 (14.08%)	1/718 (0.14%)	-	25/610 (4.1%)	4/610 (0.66%)
Liver	177/3513 (5.04%)	2/663 (0.3%)	-	21/373 (5.63%)	2/373 (0.54%)
Lung	805/16,563 (4.86%)	187/1006 (18.59%)	-	329/1019 (32.29%)	8/1019 (0.79%)
– adenocarcinoma	295/7542 (3.91%)	6/375 (1.6%)	-	34/378 (8.99%)	5/378 (1.32%)
– non-small cell carcinoma	125/3728 (3.35%)	-	-	**-**	-
– squamous cell carcinoma	230/2832 (8.12%)	181/500 (36.2%)	-	279/502 (55.58%)	-
Meninges	100/478 (20.92%)	-	-	-	-
Esophagus	314/3866 (8.12%)	21/510 (4.12%)	-	45/125 (36%)	1/125 (0.8%)
Ovary	531/4615 (11.51%)	58/684 (8.48%)	-	131/266 (29.25%)	-
– clear cell carcinoma	254/763 (33.29%)	-	-	-	-
– endometrioid carcinoma	79/327 (24.16%)	-	-	-	-
– serous carcinoma	52/1634 (3.18%)	58/568 (10.21%)	-	131/266 (49.25%)	-
Pancreas	122/3893 (3.13%)	1/898 (0.11%)	-	8/179 (4.47%)	2/179 (1.12%)
Penis	38/233 (16.31%)	-	-	-	-
Peritoneum	7/188 (3.72%)	-	-	-	-
Pituitary	12/467 (2.57%)	-	-	-	-
Pleura	7/639 (1.1%)	1/87 (1.15%)	1/87 (1.15%)	-	-
Prostate	296/4124 (7.18%)	5/949 (0.53%)	-	15/498 (3.01%)	-
Salivary gland	89/707 (12.59%)	-	-	-	-
Skin	378/4887 (7.73%)	1/587 (0.17%)	1/587 (0.17%)	24/473 (5.07%)	
Small intestine	24/434 (5.53%)	-	-	-	-
Soft tissue	288/3718 (7.75%)	-	-	12/263 (4.56%)	-
Stomach	484/4584 (10.56%)	13/472 2.75%)	-	23/285 (8.07%)	-
Testis	10/628 (1.59%)	2/149 (1.34%)	-	-	-
Thymus	8/442 (1.81%)	-	-	-	-
Thyroid	210/5263 (3.99%)	1/490 (0.2%)	-	17/513 (3.31%)	3/513 (0.58%)
Upper aerodigestive tract	507/4898 (10.35%)	75/520 (14.42%)	-	142/522 (27.2%)	3/522 (0.57%)
Urinary tract	552/2732 (20.2%)	11/399 (2.76%)	-	39/408 (9.56%)	3/408 (0.74%)
Vulva	27/142 (19.01%)	^-^	-	-	-

^1^ Mutated samples/total number of analyzed samples (%); -, no data. The data were derived from Catalogue Of Somatic Mutations in Cancer (COSMIC) v92 (released 27-AUG-20). ER, estrogen receptor; HER, human epidermal growth factor receptor; PR, progesterone receptor.

**Table 2:** Genetic alterations of the *AKT1* gene in human cancers.

Primary Tumor Tissues	Mutations (%) ^1^	Copy Number Variations (%) ^1^		Gene Expression (%) ^1^	
		Gain	Loss	Over	Under
Adrenal gland	0/790 (0%)	-	-	9/79 (11.39%)	-
Autonomic ganglia	0/1442 (0%)	-	-	-	-
Biliary tract	20/1898 (1.05%)	-	-	-	-
Bone	16/890 (1.8%)	-	-	-	-
Breast	490/12,455 (3.93%)	5/1492 (0.34%)	2/1492 (0.13%)	69/1104 (6.25%)	3/1104 (0.27%)
– ductal carcinoma	186/3743 (4.97%)	-	-	-	-
– ER-positive carcinoma	71/1475 (4.81%)	-	-	-	-
– HER-positive carcinoma	1/301 (0.33%)	-	-	-	-
– basal (triple-negative) carcinoma	9/553 (1.63%)	-	-	-	-
– ER-PR-positive carcinoma	22/364 (6.04%)	-	-	-	-
Central nervous system	15/4711 (0.32%)	4/1035 (0.39%)	1/1035 (0.1%)	22/697 (3.16%)	43/697 (6.17%)
Cervix	11/901 (1.22%)	1/299 (0.33%)	-	15/307 (4.89%)	7/307 (2.28%)
Endometrium	58/1575 (3.68%)	2/586 (0.34%)	-	49/602 (8.14%)	6/602 (1%)
– endometrioid carcinoma	44/954 (4.61%)	2/530 (0.38%)	-	39/545 (7.16%)	6/545 (1.1%)
Eye	1/370 (0.27%)	-	-	-	-
Gastrointestinal tract	0/1 (0%)	-	-	-	-
Hematopoietic and lymphoid	33/8040 (0.41%)	-	1/661 (0.15%)	7/221 (3.17%)	2/221 (0.9%)
Kidney	10/3008 (0.33%)	-	4/995 (0.4%)	26/600 (4.33%)	27/600 (4.5%)
Large intestine	147/8066 (1.82%)	-	-	18/610 (2.95%)	44/610 (7.21%)
Liver	31/2822 (1.1%)	1/663 (0.15%)	1/663 (0.15%)	24/373 (6.43%)	24/373 (6.43%)
Lung	96/11467 (0.84%)	10/1006 (0.99%)	2/1006 (0.2%)	89/1019 (8.73%)	46/1019 (4.51%)
– adenocarcinoma	41/6799 (0.6%)	4/375 (1.07%)	-	44/378 (11.64%)	12/378 (3.17%)
– non-small cell carcinoma	4/644 (0.62%)	-	-	-	-
– squamous cell carcinoma	18/2138 (0.84%)	6/500 (1.2%)	-	37/502 (7.37%)	26/502 (5.18%)
Meninges	269/1766 (15.23%)	-	-	-	-
Esophagus	14/2601 (0.54%)	1/510 (0.2%)	-	9/125 (7.2%)	-
Ovary	32/2012 (1.59%)	8/684 (1.17%)	-	33/266 (12.41%)	6/266 (2.26%)
Pancreas	29/3155 (0.92%)	-	-	10/179 (5.59%)	8/179 (4.47%)
Penis	1/101 (0.99%)	-	-	-	-
Peritoneum	3/201 (1.49%)	-	-	-	-
Pituitary	0/88 (0%)	-	-	-	-
Pleura	1/511 (0.2%)	-	-	-	-
Prostate	161/4202 (3.83%)	-	-	22/498 (4.42%)	5/498 (1%)
Salivary gland	7/535 (1.31%)	-	-	-	-
Skin	128/3730 (3.43%)	-	-	21/473 (4.44%)	-
Small intestine	2/315 (0.63%)	-	-	-	-
Soft tissue	35/2336 (1.5%)	1/264 (0.38%)	-	28/263 (10.65%)	2/263 (0.76%)
Stomach	42/3628 (1.16%)	-	-	15/285 (5.26%)	10/285 (3.51%)
Testis	2/602 (0.33%)	1/149 (0.67%)	-	-	-
Thymus	1/379 (0.26%)	-	-	-	-
Thyroid	39/3657 (1.07%)	-	-	24/513 (4.68%)	1/513 (0.19%)
Upper aerodigestive tract	27/2621 (1.03%)	2/520 (0.38%)	-	63/522 (12.07%)	23/522 (4.41%)
Urinary tract	47/1749 (2.69%)	-	2/399 (0.5%)	21/408 (5.15%)	12/408 (2.94%)
Vulva	0/64 (0%)	-	-	-	-

^1^ Mutated samples/total number of sample analyzed (%); -, no data. The data were derived from COSMIC v92 (released 27-AUG-20). ER, estrogen receptor; HER, human epidermal growth factor receptor; PR, progesterone receptor.

**Table 3:** Genetic alterations of *EGFR* in human cancers.

Primary Tumor Tissues	Mutations (%) ^1^	Copy Number Variations (%) ^1^		Gene Expression (%) ^1^	
		Gain	Loss	Over	Under
Adrenal gland	14/1112 (1.26%)	-	1/267 (0.37%)	4/79 (5.06%)	-
Breast	304/10,708 (2.84%)	14/1492 (0.94%)	2/1492 (0.13%)	65/1104 (5.89%)	-
– ductal carcinoma	157/3346 (4.69%)	-	-	-	-
– ER-positive carcinoma	8/466 (1.72%)	-	-	-	-
– HER-positive carcinoma	33/609 (5.42%)	-	-	-	-
– basal (triple-negative) carcinoma	19/1182 (1.61%)	-	-	-	-
– ER-PR-positive carcinoma	2/312 (0.64%)	-	-	-	-
Central nervous system	581/6021 (9.65%)	280/1035 (27.05%)	-	142/697 (20.37%)	-
Cervix	15/1038 (1.45%)	6/299 (2.01%)	-	27/307 (8.79%)	-
Endometrium	91/1432 (6.35%)	3/586 (0.51%)	-	41/602 (6.81%)	-
– endometrioid carcinoma	51/819 (6.23%)	3/530 (0.57%)	-	41/545 (7.52%)	-
Hematopoietic and lymphoid	193/8438 (2.29%)	1/661 (0.15%)	1/661 (0.15%)	18/221 (8.14%)	-
Kidney	56/3554 (1.58%)	2/995 (0.2%)	3/995 (0.3%)	20/600 (3.33%)	-
Large intestine	345/9946 (3.47%)	13/718 (1.81%)	1/718 (0.14%)	61/610 (10%)	5/610 (0.82%)
Liver	225/3015 (7.46%)	2/663 (0.3%)	-	20/373 (5.36%)	-
Lung	26,499/99,694 (26.58%)	53/1006 (5.27%)	1/1006 (0.1%)	148/1019 (14.52%)	-
– adenocarcinoma	14,832/48,449 (30.61%)	14/375 (3.73%)	-	58/378 (15.34%)	-
– non-small cell carcinoma	9016/35,920 (25.1%)	-	-	-	-
– squamous cell carcinoma	413/5824 (7.09%)	32/500 (6.4%)	1/500 (0.2%)	65/502 (12.95%)	-
Meninges	150/394 (38.07%)	-	-	-	-
Esophagus	134/3500 (3.83%)	15/510 (2.94%)	1/510 (0.2%)	26/125 (20.08%)	-
Ovary	89/2481 (3.59%)	2/684 (0.29%)	1/684 (0.15%)	26/266 (9.77%)	-
Pancreas	136/3681 (3.69%)	-	1/898 (0.11%)	4/179 (2.23%)	-
Prostate	282/3949 (7.14%)	1/949 (0.11%)	1/949 (0.11%)	23/498 (4.62%)	-
Skin	242/3858 (6.27%)	4/587 (0.68%)	-	10/473 (2.11%)	-
Soft tissue	66/2966 (2.23%)	3/264 (1.14%)	2/264 (0.76%)	30/263 (11.41%)	-
Stomach	146/3335 (4.38%)	19/472 (4.03%)	2/472 (0.42%)	33/285 (11.58%)	1/285 (0.35%)
Thyroid	33/3623 (0.91%)	-	-	27/513 (5.26%)	-
Upper aerodigestive tract	153/4981 (3.07%)	44/520 (8.46%)	1/520 (0.19%)	78/522 (14.94%)	-
Urinary tract	51/1649 (3.09%)	19/399 (4.76%)	1/399 (0.25%)	42/408 (10.29%)	-

^1^ Affected samples/total number of samples analyzed (%); -, no data. The data were derived from COSMIC v92 (released 27-AUG-20). ER, estrogen receptor; HER, human epidermal growth factor receptor; PR, progesterone receptor.

**Table 4:** Genetic alterations of *PTEN* in human cancers.

Primary Tumor Tissues	Mutations (%) ^1^	Copy Number Variations (%) ^1^		Gene Expression (%) ^1^	
		Gain	Loss	Over	Under
Adrenal gland	1/816 (0.12%)	-	-	6/79 (7.59%)	2/79 (2.53%)
Autonomic ganglia	3/1404 (0.21%)	-	-	-	-
Biliary tract	92/1922 (4.81%)	-	-	-	-
Bone	11/889 (1.24%)	-	-	-	-
Breast	567/10,325 (5.49%)	1/1492 (0.07%)	31/1492 (2.08%)	21/1104 (1.9%)	47/1104 (4.26%)
– ductal carcinoma	214/2832 (7.56%)	-	-	-	-
– ER-positive carcinoma	94/1477 (6.36%)	-	-	-	-
– HER-positive carcinoma	11/275 (4%)	-	-	-	-
– basal (triple-negative) carcinoma	21/469 (4.48%)	-	-	-	-
– ER-PR-positive carcinoma	12/403 (2.98%)	-	-	-	-
Central nervous system	1040/7783 (13.36%)	-	46/1035 (4.44%)	19/697 (2.73%)	146/679 (20.95%)
Cervix	74/1598 (4.63%)	-	12/299 (4.01%)	13/307 (4.23%)	21/307 (6.84%)
Endometrium	1565/3944 (39.68%)	1/586 (0.17%)	19/586 (3.24%)	50/602 (8.31)	37/602 (6.15%)
– endometrioid carcinoma	1170/2100 (55.71%)	1/530 (0.19%)	17/530 (3.21%)	34/545 (6.24%)	27/545 (4.95%)
Eye	15/401 (3.74%)	-	-	-	-
Hematopoietic and lymphoid	388/14,559 (2.67%)	-	-	9/221 (4.07%)	1/221 (0.45%)
Kidney	132/3451 (3.82%)	-	4/995 (0.4%)	19/600 (3.17%)	33/600 (5.5%)
Large intestine	476/8913 (5.34%)	-	11/718 (1.53%)	14/610 (2.3%)	46/610 (7.54%)
Liver	184/2979 (6.18%)	1/663 (0.15%)	10/663 (1.51%)	10/373 (2.68%)	13/373 (3.49%)
Lung	319/9054 (3.52%)	1/1006 (0.1%)	21/1006 (2.09%)	28/1019 (2.75%)	83/1019 (8.15%)
– adenocarcinoma	94/4362 (2.15%)	-	2/375 (0.53%)	13/378 (3.44%)	23/378 (6.08%)
– non-small cell carcinoma	22/1521 (1.45%)	-	-	-	-
– squamous cell carcinoma	113/1554 (7.27%)	1/500 (0.2%)	18/500 (3.6%)	12/502 (2.39%)	57/502 (11.35%)
Meninges	30/513 (5.85%)	-	-	-	-
Esophagus	63/2117 (2.98%)	-	4/510 (0.78%)	9/125 (7.2%)	10/125 (8%)
Ovary	141/2831 (4.98%)	2/684 (0.29%)	13/684 (1.9%)	7/266 (2.63%)	47/266 (17.67%)
Pancreas	103/3192 (3.23%)	1/898 (0.11%)	1/898 (0.11%)	11/179 (6.15%)	4/179 (2.23%)
Penis	2/74 (2.7%)	-	-	-	-
Peritoneum	1/225 (0.44%)	-	-	-	-
Pituitary	0/89 (0%)	-	-	-	-
Pleura	2/530 (0.38%)	-	1/87 (1.15%)	-	-
Prostate	393/4517 (8.7%)	1/949 (0.11%)	71/949 (7.48%)	16/498 (3.21%)	91/498 (18.27%)
Salivary gland	22/623 (3.53%)	-	-	-	-
Skin	309/3679 (8.4%)	-	28/587 (4.77%)	17/473 (3.59%)	30/473 (6.34%)
Small intestine	2/326 (0.61%)	-	-	-	-
Soft tissue	59/2514 (2.35%)	-	14/264 (5.3%)	5/263 (1.9%)	15/263 (5.7%)
Stomach	130/2980 (4.36%)	-	15/472 (3.18%)	7/285 (2.46%)	26/285 (9.12%)
Testis	7/639 (1.1%)	-	1/149 (0.67%)	-	-
Thymus	0/372 (0%)	-	-	-	-
Thyroid	110/3936 (2.79%)	-	1/490 (0.2%)	25/513 (4.87%)	9/513 (1.75%)
Upper aerodigestive tract	126/3441 (3.66%)	-	12/520 (2.31%)	17/522 (3.26%)	16/522 (3.07%)
Urinary tract	45/1614 (2.79%)	1/399 (0.25%)	8/399 (2.01%)	7/408 (1.72%)	20/408 (4.9%)
Vulva	12/157 (7.64%)	-	-	-	-

^1^ Mutated samples/total number of sample analyzed (%); -, no data. The data were derived from COSMIC v92 (released 27-AUG-20).

**Table 5:** Genetic alterations of *MTOR* in human cancers.

Primary Tumor Tissues	Mutations (%) ^1^	Copy Number Variations (%) ^1^		Gene Expression (%) ^1^	
		Gain	Loss	Over	Under
Adrenal gland	0/658 (0%)	-	4/267 (1.5%)	1/79 (1.27%)	-
Autonomic ganglia	0/1225 (0%)	-	-	-	-
Biliary tract	51/1388 (3.67%)	-	-	-	-
Bone	3/725 (0.41%)	-	-	-	-
Breast	258/7666 (3.37%)	1/1492 (0.07%)	2/1492 (0.13%)	39/1104 (3.53%)	32/1104 (2.9%)
– ductal carcinoma	120/2483 (4.83%)	-	-	-	-
– ER-positive carcinoma	29/1363 (2.13%)	-	-	-	-
– HER-positive carcinoma	8/205 (3.9%)	-	-	-	-
– basal (triple-negative) carcinoma	5/372 (1.34%)	-	-	-	-
ER-PR-positive carcinoma	6/308 (1.95%)	-	-	-	-
Central nervous system	56/3499 (1.6%)	1/1035 (0.1%)	-	21/697 (3.01%)	91/697 (13.06%)
Cervix	21/388 (5.41%)	-	-	18/307 (5.86%)	5/307 (1.63%)
Endometrium	103/988 (10.43%)	-	-	27/602 (4.49%)	9/602 (1.5%)
– endometrioid carcinoma	82/649 (12.63%)	-	-	23/545 (4.22%)	-
Eye	3/179 (1.68%)	-	-	-	-
Hematopoietic and lymphoid	118/6030 (1.96%)	-	-	2/221 (0.9%)	5/221 (2.26%)
Kidney	165/2998 (5.5%)	-	-	20/600 (3.33%)	12/600 (2%)
Large intestine	344/4526 (7.6%)	-	1/718 (0.14%)	16/610 (2.62%)	35/610 (5.74%)
Liver	175/2381 (7.35%)	-	-	5/373 (1.34%)	-
Lung	228/4930 (4.62%)	3/1006 (0.3%)	2/1006 (0.2%)	44/1019 (4.32%)	17/1019 (1.67%)
– adenocarcinoma	126/2724 (4.63%)	1/375 (0.27%)	1/375 (0.27%)	23/378 (6.08%)	12/378 (3.17%)
– non-small cell carcinoma	4/124 (3.23%)	-	-	-	-
– squamous cell carcinoma	44/1004 (4.38%)	1/500 (0.2%)	1/500 (0.2%)	11/502 (2.19%)	5/502 (1%)
Meninges	57/306 (18.63%)	-	-	-	-
Esophagus	79/1673 (4.72%)	1/510 (0.2%)	-	5/125 (4%)	-
Ovary	63/1396 (4.51%)	1/684 (0.15%)	-	26/266 (9.77%)	11/266 (4.14%)
Pancreas	83/2505 (3.31%)	-	-	6/179 (3.35%)	10/179 (5.59%)
Penis	2/17 (11.76%)	-	-	-	-
Peritoneum	1/43 (2.33%)	-	-	-	-
Pituitary	0/89 (0%)	-	-	-	-
Pleura	0/470 (0%)	-	-	-	-
Prostate	217/3317 (6.54%)	-	1/949 (0.11%)	14/498 (2.81%)	-
Salivary gland	7/410 (1.71%)	-	-	-	-
Skin	236/2248 (10.5%)	2/587 (0.34%)	-	39/473 (8.25%)	24/473 (5.07%)
Small intestine	5/252 (1.98%)	-	-	-	-
Soft tissue	18/1489 (1.21%)	-	-	-	-
Stomach	124/1912 (6.49%)	2/472 (0.42%)	-	18/285 (6.32%)	4/285 (1.4%)
Testis	9/662 (1.36%)	-	-	-	-
Thymus	0/173 (0%)	-	-	-	-
Thyroid	42/2131 (1.97%)	-	-	20/513 (3.9%)	7/513 (1.36%)
Upper aerodigestive tract	44/1836 (2.4%)	-	-	26/522 (4.98%)	5/522 (0.96%)
Urinary tract	72/1209 (5.96%)	-	-	34/408 (8.33%)	2/408 (0.49%)
Vulva	1/30 (3.33%)	-	-	-	-

^1^ Mutated samples/total number of sample analyzed (%); -, no data. The data were derived from COSMIC v92 (released 27-AUG-20).

**Table 6:** AKT inhibitors in phase III clinical trials ^1^.

Inhibitor	Target	Condition or Disease	ClinicalTrials.gov Identifier
Ipatasertib and Paclitaxel	AKT and microtubules	Locally advanced or metastatic TNBC; locally advanced or metastatic HR+/HER2– breast adenocarcinoma	NCT03337724 Study start date: January 2018 Estimated study completion date: December 2021
Ipatasertib and Abiraterone	AKT and CYP17	Metastatic castrate-resistant prostate cancer	NCT03072238 Study start date: June 2017 Estimated study completion date: November 2023
Ipatasertib, Palbociclib, and Fulvestrant	AKT, CDK4/6, and ER	HR+ and HER2– locally advanced unresectable or metastatic breast cancer	NCT04060862 Study start date: November 2019 Estimated study completion date: January 2026
Ipatasertib, Paclitaxel, and Atezolizumab	AKT, microtubules, and PD-L1	Locally advanced or metastatic TNBC	NCT04177108 Study start date: November 2019 Estimated study completion date: October 2025
Ipatasertib and Fulvestrant	AKT and ER	Advanced HER2– and ER+ breast cancer	NCT04650581 Estimated study start date: December 2020 Estimated study completion date: December 2026
Capivasertib and Paclitaxel	AKT and microtubules	Locally advanced or metastatic TNBC	NCT03997123 Study start date: June 2019 Estimated study completion date: January 2023
Capivasertib and Abiraterone	AKT and CYP17	Hormone-sensitive prostate cancer	NCT04493853 Study start date: July 2020 Estimated study completion date: November 2025
Capivasertib and Fulvestrant	AKT and ER	Locally advanced (inoperable) or metastatic HR+/HER2– breast cancer	NCT04305496 Study start date: April 2020 Estimated study completion date: July 2024

^1^ Clinical trial data obtained from https://clinicaltrials.gov at 25 December 2020. CDK, cyclin-dependent kinase; HER2–, human epidermal growth factor receptor 2 negative; HR+, hormone receptor positive; ER+, estrogen receptor positive; TNBC, triple-negative breast cancer.

**Table 7:** Allosteric mTOR inhibitors approved by the Food and Drug Administration (FDA) for human cancers.

Inhibitor (Trade Name)	Target	Indications	Approval Date
Sirolimus (Rapamune)	mTORC1	Treatment of patients with lymphangioleiomyomatosis	August 2000
Temsirolimus (Torisel)	mTORC1	Treatment of advanced renal cell carcinoma	May 2007
Everolimus (Afinitor)	mTORC1	- Treatment of advanced renal cell carcinoma - Treatment of advanced HR+ breast cancer - Treatment of progressive neuroendocrine tumors of pancreatic origin (PNET), of gastrointestinal (GI) or lung origin - Treatment of renal angiomyolipoma and tuberous sclerosis complex (TSC) - Treatment of subependymal giant cell astrocytoma (SEGA) associated with tuberous sclerosis (TSC)	March 2009 August 2012 February 2016

**Table 8:** Other mTOR inhibitors in Phase 2/3 clinical trials ^1^.

Inhibitor	Target	Phase	Condition or Disease	ClinicalTrials.gov Identifier
**Allosteric**				
Ridaforolimus	mTORC1	3	Metastatic soft-tissue sarcomas and metastatic bone sarcomas	NCT00538239 Study start date: October 2007 Study completion date: December 2012
	mTORC1	2	Advanced sarcoma	NCT00093080 Study start date: October 2004 Study completion date: November 2008
	mTORC1	2	Relapsed or refractory hematologic malignancies	NCT00086125 Study start date: June 2004 Study completion date: June 2006
	mTORC1	2	Endometrial cancer	NCT00122343 Study start date: August 2005 Study completion date: January 2008
	mTORC1	2	Breast cancer	NCT00736970 Study start date: July 2008 Study completion date: May 2011
**ATP-Competitive**				
MLN0128	mTORC1/2	2	Metastatic castration-resistant prostate cancer	NCT02091531 Study start date: March 2014 Study completion date: October 2018
	mTORC1/2	2	Metastatic anaplastic thyroid cancer	NCT02244463 Study start date: July 2015 Estimated study completion date: January 2022
CC-223	mTORC1/2	1/2	Multiple Myeloma Diffuse large B-cell lymphoma Glioblastoma multiforme Hepatocellular carcinoma non-small cell lung cancer Neuroendocrine tumors of non-pancreatic origin Hormone receptor-positive breast cancer	NCT01177397 Study start date: July 2010 Study completion date: December 2016
Sapanisertib (TAK-228 or MLN0128)	mTORC1/2	2	Estrogen receptor-positive breast cancer	NCT02988986 Study start date: April 2017 Study completion date: March 2019
	mTORC1/2	2	Soft-tissue sarcoma	NCT02987959 Study start date: February 2017 Estimated study completion date: September 2020
	mTORC1/2	2	Locally advanced or metastatic bladder cancer	NCT03047213 Study start date: December 2016 Estimated study completion date: June 2021
Vistusertib (AZD2014)	mTORC1/2	2	Progressive or symptomatic meningioma	NCT02831257 Study start date: August 2016 Study completion date: October 2020
	mTORC1/2	2	Estrogen receptor positive breast cancer	NCT02216786 Study start date: January 2014 Estimated study completion date: July 2020
	mTORC1/2	2	Meningioma	NCT03071874 Study start date: October 2017 Estimated study completion date: July 2024
**Dual PI3K/mTOR**				
Dactolisib (BEZ235)	PI3K/mTOR	2	Pancreatic neuroendocrine tumors (pNET)	NCT01658436 Study start date: November 2012 Study completion date: July 2015
Samotolisib (LY3023414)	PI3K/mTOR	2	Metastatic castration resistant prostate cancer	NCT02407054 Study start date: April 2015 Study completion date: April 2020
Bimiralisib (PQR309)	PI3K/mTOR	2	Primary central nervous system lymphoma	NCT02669511 Study start date: November 2015 Study completion date: January 2018
	PI3K/mTOR	2	Relapsed or refractory lymphoma	NCT02249429 Study start date: May 2015 Study completion date: September 2018
Gedatolisib(PKI587) and Talazoparib	PI3K/mTORandPARP	2	Triple-Negative Breast Cancer	NCT03911973 Study start date: April 2019 Estimated study completion date: May 2022
Apitolisib (GDC-0980)	PI3K/mTOR	2	Prostate Cancer	NCT01485861 Study start date: January 2019 Estimated study completion date: April 2021
Voxtalisib (SAR245409, XL765)	PI3K/mTOR	2	Ovarian Cancer	NCT01936363 Study start date: September 2013 Study completion date: November 2017
	PI3K/mTOR	2	Lymphoma	NCT01403636 Study start date: October 2011 Study completion date: September 2014
Paxalisib (GDC-0084)	PI3K/mTOR	2	Glioblastoma	NCT03522298 Study start date: May 2018 Estimated study completion date: December 2020

^1^ Clinical trial data obtained from https://clinicaltrials.gov at 25 December 2020.

## Data Availability

Not applicable.

## Abbreviations

AICAR	5-aminoimidazole-4-carboxamide-1-β-4-ribofuranoside
AMP	adenosine monophosphate
AMPK	AMP-activated protein kinase
BAD	BCL2-associated agonist of cell death
CTMP	carboxyl-terminal modulator protein
DEPTOR	DEP domain-containing mTOR-interacting protein
DNA-PK	DNA-dependent protein kinase
eIF4E	eukaryotic translation initiation factor 4E
ERK	extracellular signal-regulated kinase
FDA	Food and Drug Administration
FKBP12	FK506-binding protein 12
FoxO	forkhead box O transcription factors
FRB	FKBP12-rapamycin-binding
GAP	GTPase activating protein
GβL	G protein β-subunit-like protein
GSK3	glycogen synthase kinase 3
IGF-1	insulin-like growth factor-1
INPP4B	inositol polyphosphate 4-phosphatase type II
IRS-1	insulin receptor substrate 1
MAP kinase	mitogen-activated protein kinase
mLST8	mammalian lethal with SEC13 protein 8
mSIN1	stress-activated map kinase-interacting protein 1
mTOR	mechanistic/mammalian target of rapamycin
PDCD4	programmed cell death protein 4
PDK1	phosphoinositide-dependent kinase 1
PI3K	phosphatidylinositol-3-kinase
PIKK	phosphatidylinositol 3-kinase-related kinase
PI(4,5)P2	phosphatidylinositol 4,5 bisphosphate
PI(3,4,5)P3	phosphatidylinositol 3,4,5 trisphosphate
PLD	phospholipase D
PRAS40	proline-rich AKT1 substrate 1
Protor	protein observed with RICTOR
PTEN	phosphatase and tensin homolog
RAPTOR	regulatory-associated protein of mTOR
Rheb	Ras homolog enriched in brain
RICTOR	rapamycin insensitive companion of mTOR
RSK	ribosomal S6 kinase
RTK	receptor tyrosine kinase
S6K1	p70 S6 kinase, ribosomal protein S6 kinase beta-1
SGK1	serum and glucocorticoid-activated kinase 1
SHIP	Src homology 2 (SH2) domain containing inositol polyphosphate 5-phosphatase
TBC1D7	TBC1 domain family member 7
TIF-1A	transcription initiation factor 1A
TSC	tuberous sclerosis complex
UBF	upstream binding factor
